# The Collaborative Study on the Genetics of Alcoholism

**Published:** 1995

**Keywords:** government agency, research, AOD dependence, genetics and heredity, molecular genetics, genetic mapping, phenotype, genotype, biological markers, evoked potential, dose response relationship, screening and diagnostic method for AODD (alcohol and other drug use disorders), data collection, data analysis method

## Abstract

In 1989 the National Institute on Alcohol Abuse and Alcoholism initiated the Collaborative Study on the Genetics of Alcoholism (COGA), a large-scale, multidisciplinary research program to investigate the genetic components of the susceptibility to alcohol abuse and dependence. COGA involves six research centers located across the United States. The following articles by leading COGA investigators provide an overview of the design of this study and its components and of the challenges inherent to an endeavor of this scope. The authors also present some of the results obtained through COGA to date. Although sometimes still preliminary in nature, these findings reflect COGA’s potential for greatly improving our knowledge of the complex disorder of alcoholism.

COGA is a unique and comprehensive research effort whose goal is to elucidate the genetic mechanisms that contribute to a person’s susceptibility to alcohol abuse and dependence. Many scientists from different research areas are collaborating to assess hundreds of families and their phenotypes^1^ related to alcoholism and associated behaviors (e.g., alcohol consumption and the presence of psychiatric disorders) and to correlate these phenotypes with genetic characteristics. Because no single group of investigators in the alcohol research field has the expertise and experience necessary for a study of this scope, a consortium of scientists is designing and implementing the investigation.

## The Historic Background of COGA

During the past 25 years, a vast array of human studies has supported the theory of a genetic component in the susceptibility to alcoholism. For example, family studies measuring the prevalence of alcoholism and related disorders consistently found that biological relatives of people who abused or were dependent on alcohol were at a significant risk of developing the disorder themselves (Cotton 1979). The degree of risk generally correlated with the degree of the relationship (i.e., the closer the relationship, the higher the risk). A person’s risk for alcoholism also correlated with the frequency of the disorder in his or her family (i.e., the more alcoholic relatives, the higher the risk). Despite these consistent findings, however, it has been difficult to compare the individual studies because the selection of families, the criteria for a diagnosis of alcoholism, and the assessment methods rarely were standardized.

In addition to family studies, researchers have used analyses of identical, or monozygotic (MZ), and fraternal, or dizygotic (DZ), twins to measure the heritability of alcoholism. Studies of Scandinavian and American twins found a substantial genetic influence. Similarly, twin studies evaluated the contribution of genetic factors to alcohol consumption. Although the estimates of the heritability of alcohol consumption varied, most studies identified some genetic contribution, albeit to a lesser extent than for alcoholism.

One American and two Scandinavian adoption studies also provided evidence that powerful genetic factors play an important role in the familial transmission of alcoholism (Hesselbrock 1995). Adoption studies allow researchers to separate genetic and environmental influences on the development of alcoholism and related psychopathology; the studies also can correlate the extent to which alcoholism is transmitted to characteristics of the biological parents and the postnatal environment. Accordingly, adoption studies not only have provided conclusive evidence for large genetic effects but also have allowed a heuristically valuable, genetics-based subdivision of alcoholism.

Cloninger and colleagues (Cloninger et al. 1978) used a large-scale adoption study in Sweden to identify genetic and other variables that predicted alcohol abuse in adoptees. The study distinguished two independent types of alcoholism. Type I alcoholism usually developed after age 25, occurred in both males and females, and was marked by frequent psychological dependence as well as guilt and fear about alcohol dependence. In contrast, type II alcoholism developed before age 25, generally was limited to men, and frequently was marked by spontaneous alcohol seeking as well as aggressive behavior (e.g., fighting and arrests) when drinking. Type II alcoholism had a greater genetic component than type I alcoholism.

## Characteristics of COGA

As indicated by the example above, alcohol abuse and dependence are extremely variable phenomena, and more than one genetic mechanism probably contributes to their development. To facilitate the detection of specific genetic mechanisms, the COGA investigators therefore decided to pursue a multidimensional approach to assess their subjects’ phenotypes and to ensure a careful dissection of various clinical aspects of the disease. Consequently, the study seeks to measure a variety of variables that have been correlated with familial or genetic risk factors for alcoholism. For example, COGA analyses include neurophysiological measures, such as certain kinds of brain waves that have been shown to be abnormal in sons of alcoholic fathers (Begleiter et al. 1984).

COGA researchers also extensively use powerful new genetic tools to identify genes that play a major role in the susceptibility to alcoholism. During the past 5 years, such tools have allowed great strides in the study of complex familial disorders, such as schizophrenia and alcoholism, in which significant and different genetic factors interact with cultural and personal variables. For example, highly polymorphic DNA markers (i.e., DNA fragments that exist in many different forms and vary widely among persons) that are closely spaced throughout the genome now are broadly available and have enhanced greatly the potential for conducting genetic linkage studies.

## Outlook

The search for genes that modify a person’s susceptibility to alcohol abuse and dependence is a central concern of alcohol studies. With the powerful and reliable assessment tools now available, COGA ultimately will identify one or more such genes. Scientists have identified biological markers that strongly correlate with the familial transmission of alcoholism, and the new tools of molecular genetics make detailed genetic linkage studies possible. The discovery of a genetic linkage between alcoholism and a known genetic marker would be an important first step in the systematic search for the genetic mechanisms underlying the disease. With its comprehensive approach, COGA will greatly increase our understanding of this complex biological, psychological, and social disorder.

## Figures and Tables

**Table 1:** P300 Characteristics of Stage II Subjects and Control Subjects in the COGA Study

	Mean P300 Amplitude (microvolts [*μ*V]) -		Subjects With P300 ≥ 2 SDs^1^ Below the Mean (%)	
		
	Stage II	Control	Stage II	Control
				
All	17.4	21.2	10.0	1.1
Alcoholic	15.9	21.2	22.1	2.9
Nonalcoholic	17.5	20.4	6.8	1.1

1 SD = standard deviation.
