# Supplementary material for: The Collaborative Study on the Genetics of Alcoholism
Source: Alcohol Health Res World. 1995;19(3):228–36. (PMC6875768)
Supplement: Related texts

# Research article: DATA COLLECTION

**Published:** 1995

**Authors:** Theodore Reich

**Affiliations:** Theodore Reich, M.D., is a professor in the Department of Psychiatry, Washington University School of Medicine, St. Louis, Missouri

COGA’s ultimate goal is to detect and map (i.e., determine the location on the genome) genes that contribute to a person’s susceptibility to alcohol dependence and other alcohol-related phenotypes by studying the transmission of alcoholism in large families with several alcoholic members. To achieve this goal, COGA investigators have designed a comprehensive research strategy, the ascertainment protocol, that divides the analyses into several stages. This approach is necessary because COGA’s scope requires collecting and analyzing a large sample of suitable families from across the country. Furthermore, because six research centers are involved in the study, the ascertainment protocol must ensure that all data are comparable among the centers.

The study design also must take into account that the diagnosis “alcohol dependence” encompasses a variety of syndromes and includes abnormalities in many domains of a person’s behavior and physiological functioning. This diversity necessitates a wide range of assessment procedures, including conducting comprehensive personal interviews; evaluating personality traits, neuropsychological variables, and biochemical markers; and obtaining and evaluating neurophysiological measurements. These procedures are followed by the genetic analysis of families with many alcoholic members (i.e., the families have a high density of alcoholism). Such families provide important information for genetic studies in which the precise location of the genes that predispose a person to alcohol dependence is being investigated. Finally, the study must include control families to provide base rates for many of these variables in the general population.

## Selection of Study Subjects

The initial study subjects (i.e., the probands) are randomly selected clients in outpatient and inpatient alcohol treatment facilities. The probands must not be intravenous drug users, HIV positive, or terminally ill. They must be able to participate in the interview process and provide written informed consent for their own and their families’ participation in the study. In addition, the proband’s family must be large and complete enough to be informative for a detailed genetic study, and at least two first-degree relatives must live in one of the catchment areas covered by the six COGA centers. To be classified as alcoholic, probands and other affected relatives must meet the criteria for alcohol dependence of the *Diagnostic and Statistical Manual of Mental Disorders, Third Edition, Revised* (DSM–III–R) (American Psychiatric Association [APA] 1987) and the criteria established by Feighner (1972) for “definite” alcoholism.

The control families (two parents and three or more offspring over the age of 14) are selected randomly from the community. Because the control subjects are supposed to reflect the baseline values for alcohol-related parameters in the general population, alcoholics or people with other illnesses (with the exception of HIV-positive subjects) are not excluded. All control subjects participate in a personal interview, personality tests, and neuropsychological and neurophysiological assessment. Researchers also obtain blood samples from the controls for biochemical marker studies.

## The COGA Ascertainment Protocol

COGA’s ascertainment protocol has two major components. First, it allows researchers to characterize the familial distribution of alcohol dependence and related phenotypes (e.g., alcohol consumption and coexisting psychiatric disorders) for multiple domains, thus providing the basis for selecting the quantitative and qualitative variables for genetic analysis. Second, following extensive interviews with nuclear family members (i.e., the proband and his or her spouse and first-degree relatives), the protocol includes the selection and genetic study of densely affected families, which are especially informative for linkage and association analyses.

### Stage I: Assessing the Probands and Their Families

All probands, their spouses, and their first-degree relatives who meet admission criteria are interviewed using the Semi-Structured Assessment for the Genetics of Alcoholism (SSAGA), a questionnaire that was developed specifically for use in this study. At the same time, researchers assess other personality traits in the subjects. The interviews also include children and adolescents age 7 and older using age-appropriate questionnaires (i.e., the CSSAGA–C for children ages 7 to 12 and the CSSAGA–A for adolescents ages 13 to 17). An additional questionnaire, the CSSAGA–P, is used to interview parents about their children’s psychiatric status.

The data derived from these interviews constitute the stage I sample. Because they were generated using a single, well-defined ascertainment scheme (i.e., the collection of SSAGA questionnaires), they allow COGA researchers to study the familial transmission of alcoholism.

### Stage II: In-Depth Phenotypic and Genetic Analyses

Families with three or more alcohol-dependent members according to the criteria described previously are accepted for a more detailed phenotypic and genetic study. Scientists assess all first-degree relatives of alcoholic subjects with a battery of neuropsychological tests and obtain blood samples to evaluate the presence of biochemical markers that may be associated with alcoholism. Furthermore, all adult and juvenile family members undergo a series of neurophysiological tests, including the evaluation of their electroencephalogram (EEG) and other brain waves.

In addition to evaluating these personal characteristics, researchers also establish the family history of alcohol dependence and general psychopathology for all family members. This family history complements and validates personal interview data and allows researchers to diagnose family members who are unavailable for interviews. Whenever possible, medical and psychiatric records supplement these data. For juvenile subjects, the information also includes school records and other environmental variables.

Blood samples are obtained from all family members to prepare their DNA for genetic linkage and association studies. Researchers generate tissue-culture cell lines from the blood of adult subjects that can be used to produce high-quality DNA or frozen and stored (i.e., cryopreserved) for later analyses. The blood from juvenile subjects (for whom one does not know whether they will develop alcoholism) also is cryopre-served. These samples can be used later to prepare DNA for linkage and association studies that confirm and extend earlier findings.

These detailed phenotypic and initial genetic data make up the stage II sample. They allow researchers to identify informative subsets of families for additional genetic studies.

The stage II sample families are extended further by administering the complete assessment protocol to the nuclear families of second- and third-degree relatives of some of the initial probands. This is done if the proband’s family history suggests that these extended families include two or more alcohol-dependent members, even if the connecting relative is unaffected. Similarly, if one of the proband’s first-degree relatives is alcoholic, the study will include the nuclear families of second-degree relatives of this family branch. The goal of these extensions is to establish large, densely affected, multigenerational family trees. Researchers try to avoid, however, including nuclear families with two alcohol-dependent parents in the stage II analyses in order to trace alcohol-related behavioral and genetic traits more easily.

#### The Current Status of Stage I and II

To date, COGA researchers have interviewed 5,778 adults and 674 juveniles from 1,051 families as the stage I sample, 908 adults and 177 juveniles from 204 families as the control sample, and 2,438 adults and 416 juveniles from 259 families as the stage II sample. In addition, the researchers have assessed 2,839 subjects neuropsychologically, obtained blood samples from 3,032 subjects for biological marker studies, and cryopreserved 2,288 blood cell lines.

### Stage IV: Genotyping the Subjects

The next step in the COGA protocol is the genotyping (i.e., determining the specific genotype, or genetic makeup) of the selected informative subjects and families from the stage II sample. Families are genotyped if they have a large proportion of alcohol-dependent members and if a sufficient number of DNA samples from these members are available for analyzing. These families are designated as the “stage IV” sample.

Families with members who are uncooperative or who are not informative for the study of genetic linkage are designated as the “stage III” sample and are not studied further.

Initially, researchers plan to use genetic markers spaced about 20 centimorgans (cM) apart throughout the genome to determine each subject’s genotype for each marker. If any marker appears to be linked to alcoholism, additional adjacent markers and subjects will be included in the analysis.

COGA researchers will use several different methods to determine if any linkage exists between alcohol-related phenotypes and specific genotypes, modifying these methods to accommodate the special characteristics of alcohol dependence and alcohol-related phenotypes. Scientists also will improve methods to detect associations between specific marker alleles and alcohol dependence. Additionally, the COGA design allows other investigators to reproduce reported linkages or associations.

DNA samples from about 990 informative subjects from 106 families currently are being genotyped. These stage IV samples constitute the first wave of COGA’s genotyping effort. These early results will be replicated later using a sample of similar size.

An important focus of the stage IV analysis is the evaluation of sibling pairs in which both siblings are alcohol dependent. Because siblings share 50 percent of their genetic material with each other and with their parents, the similarities and differences in genotype and behavior that exist between them are particularly informative. Using the diagnostic criteria described previously, 385 sibling pairs fall into this category. For more than 90 percent of these pairs, DNA samples also are available from at least one parent. Even using more stringent criteria to diagnose alcoholism, such as the criteria defined by the *Diagnostic and Statistical Manual of Mental Disorders, Fourth Edition* (DSM–IV), COGA includes 281 appropriate sibling pairs. DNA from at least one parent is available for genotyping for approximately 94 percent of these pairs. This sample size will enable scientists to detect genes contributing to the susceptibility for alcoholism, even if several genes interact to produce the phenotype.

Several approaches to linkage analysis are planned. First, “parametric” methods that model the underlying genetic transmission of alcohol dependence and linkage will be used. These methods include affected and unaffected relatives, often in multiple generations. Second, “nonparametric” methods, which include affected siblings and other relative pairs, will be used.

The parametric methods tend to have greater power but can only be used when the underlying mode of transmission is specified. In contrast, nonparametric models do not require the genetic transmission model to be specified. However, the nonparametric models generally are less powerful. In our study, both approaches will be used.

Many of the sibling pairs also have undergone neurophysiological evaluation. These data will allow researchers to detect genes that influence heritable EEG and other brain wave characteristics. Neurophysiological analyses have focused on families with at least three alcohol-dependent first-degree relatives. Accordingly, quantitative neurophysiological differences between members of the test families and the general population provide a measure of the subjects’ susceptibility to alcoholism. This correlation enhances researchers’ ability to identify the underlying genes through linkage analyses.

## Future Perspective

COGA investigators plan to complete their sample collection in 1996. At that time, approximately 1,400 additional informative subjects will be available for the second wave of COGA’s genotyping efforts, which will replicate the earlier results. Replication is necessary because the large number of statistical tests to be performed on the data from the first wave is expected to result in some spurious findings (i.e., false positives). The two-stage analytic protocol is the most cost-effective approach to unambiguously detect genes that individually may have small or moderate effects but combined determine a certain phenotype.

## References

[b5-arhw-19-3-228] American Psychiatric Association (1987). Diagnostic and Statistical Manual of Mental Disorders, Third Edition, Revised.

[b6-arhw-19-3-228] Feighner J, Robins E, Guze S, Woodruff R, Winokur G, Munoz R (1972). Diagnostic criteria for use in psychiatric research. Archives of General Psychiatry.

# Research article: CLINICAL ASSESSMENT

**Published:** 1995

**Authors:** Victor Hesselbrock

**Affiliations:** Victor Hesselbrock, Ph.D., is a professor in the Department of Psychiatry, University of Connecticut School of Medicine, Farmington, Connecticut

The familial nature of alcoholism is well documented. The genes that contribute to the susceptibility to alcoholism, however, and the mechanisms through which they determine the observable characteristics of the disorder (i.e., the phenotype), remain unknown. A major obstacle to clarifying the genetics of complex diseases such as alcoholism is the lack of a reliable phenotype that can be linked to a genetic marker.

Researchers usually establish phenotypes (i.e., subtypes) and the differences among them based on the clinical presentation of the people affected by the disease (i.e., the probands). The clinical picture of alcoholism, however, varies widely with respect to drinking behavior as well as associated medical and psychiatric symptoms and conditions. Other personal factors, such as temperament and cognitive style, also may influence the expression of drinking behavior. Furthermore, different family backgrounds; rearing patterns; and biological, social, and psychiatric problems have been associated with certain alcohol-use patterns. These factors may influence treatment-seeking behavior, treatment outcome, and the course of alcoholism over the life span by moderating or enhancing the genetic predisposition (Hesselbrock and Hesselbrock 1990). All these phenomena complicate issues such as defining the nature of alcoholism (e.g., separating it from normal drinking), identifying distinct boundaries between alcoholism and other psychiatric disorders, and determining differences among population subgroups.

Several subtypes of alcoholism have been proposed (cf. Jellinek 1960; Cloninger 1981; Babor et al. 1992), but it is difficult to identify possible genetic relationships for most of them. Most studies of typologies and phenotypes have focused only on a cross-sectional sample of alcoholics and therefore cannot adequately address questions relating to the numerous mechanisms that may contribute to the development of alcoholism over a person’s life span. To elucidate variations in the etiology of different alcoholism subtypes, researchers would need to compare different alcoholism phenotypes (e.g., by focusing on certain clinical or biological characteristics of affected persons) (Baron et al. 1990); examine the stability of phenotypes over time; and study their transmissibility within families. Such studies could help determine whether the phenotypes reflect actual genotypes related to the development of alcoholism or whether they result instead from a variety of social or psychological factors associated with alcoholism. Finally, stable and reliable phenotypic information, including diagnostic classification, is crucial for genetic linkage studies that are affected strongly by misclassifications (Weissman et al. 1986; Baron et al. 1990; Maziade et al. 1992).

These problems illustrate the critical role of accurate clinical assessment in genetic studies of behavioral traits. Consequently, the COGA Assessment Committee was created to identify or develop accurate, reliable, and comprehensive assessment criteria and phenotypic dimensions for COGA’s genetic and other biological studies. The committee developed an assessment protocol that examines the probands’ psychiatric factors, family environment, drinking history, personality traits, and cognitive functioning.

## The Semi-Structured Assessment for the Genetics of Alcoholism

Because none of the existing interview schedules appeared to be ideal for the study’s principal focus (i.e., the assembly of an archival data set on alcoholic families for linkage studies and multiple phenotypic measurements), the COGA investigators developed a new semistructured psychiatric diagnostic protocol. Its psychiatric assessment portion had to fulfill three central requirements: It should permit diagnosis according to multiple established criteria systems to allow data comparison with existing studies; it should permit the assessment of comorbidity with other psychiatric disorders and a chronological ordering of symptoms and syndromes; and it should encourage a systematic description of alcoholism and related phenotypes that corresponds to our clinical understanding of these conditions.

The result of this effort was the Semi-Structured Assessment for the Genetics of Alcoholism, or SSAGA, a diagnostic interview based on the DSM–III–R diagnostic system. In addition, SSAGA allows researchers to make alcohol-related diagnoses according to a variety of diagnostic systems, including the Feighner criteria, the Research Diagnostic Criteria, DSM–III, DSM–IV, and the World Health Organization’s tenth edition of the *International Classification of Diseases* (ICD–10). SSAGA also permits differential diagnoses in terms of the symptoms as well as age of onset of different conditions.

To determine SSAGA’s reliability, researchers conducted test-retest studies both within and between different COGA centers. The studies indicated good-to-high reliability for DSM–III–R-based diagnoses of substance-use disorders (e.g., alcoholism), depression, and antisocial personality disorder (Bucholz et al. 1994, 1995).

SSAGA is part of a family of diagnostic instruments covering different aspects of clinical assessment. For example, the Family History Assessment Module (F–HAM) (Rice et al. 1995) helps determine the prevalence of DSM–III–R psychiatric conditions among biological relatives, whereas the CSSAGA assesses the past and current psychiatric status of children and adolescents. These psychiatric assessment tools also are available to investigators outside of COGA and currently are being used in several different clinical investigations of substance-use disorders, psychiatric disorders, and medical conditions.

Scientists from Europe, Asia, South America, and Oceania also are using parts or all of the COGA assessment protocol in related studies of the genetics of alcoholism. French, Spanish, Portuguese, German, Chinese, and Japanese versions of SSAGA are available. This collaborative effort will result in a large data set that will permit the study of alcoholism phenotypes across a variety of ethnic and cultural populations.

## Preliminary Studies Related to Phenotyping

The growing COGA database has been used for several analyses that relate to phenotypes of alcoholism. These studies have addressed issues such as the usefulness of etiologic factors for phenotyping purposes, associated psychopathology, diagnostic system comparisons, gender differences, and different typologies.

### Time Course of Development of Alcohol Problems

Schuckit and colleagues (1995*a*) determined the age at the first occurrence of 44 alcohol-related life experiences addressed by SSAGA for 478 alcoholic (as defined by DSM–III–R) subjects (including 161 women) and for 444 drinking but nonalcoholic subjects (261 women). The order in which alcohol-related problems appeared was similar to that of another, non-COGA sample of alcoholic male inpatients. The order of appearance of problems also was similar for men and women in the COGA sample and for subjects with and without alcoholism treatment.

When the researchers examined 19 alcohol-related life experiences in the non-alcoholic drinkers, they found that the order of first occurrence was similar to that observed for alcoholic subjects. Thus, the order in which early symptoms of alcoholism occur does not appear to differ significantly among persons who eventually become affected and those who remain unaffected.

### The Prevalence of Anxiety Disorders in Relatives of Alcoholics

Previous studies have indicated a close association between anxiety symptoms, drinking behavior, and alcohol dependence. It still is unclear, however, whether the anxiety conditions are lifelong disorders or temporary organic conditions related to intoxication and withdrawal. One approach to better understanding this relationship is to observe the rate of diagnosable anxiety disorders in close biological relatives of alcoholics.

Using SSAGA, researchers interviewed 591 first-degree relatives of alcohol-dependent men and women (Schuckit 1995*b*). Focusing on anxiety disorders included in DSM–III–R, the preliminary analysis found that first-degree relatives of alcoholics had lifetime risks for panic disorder, agoraphobia, social phobia, and obsessive-compulsive disorder similar to the general population. These findings suggest that anxiety disorders are not exceptionally prevalent among close biological relatives of alcoholics and do not support a common genotype for the two disorders.

### Personality Disorders

Although COGA generally does not assess diagnosable personality disorders other than antisocial personality disorder, researchers at the study’s Iowa center also have evaluated other personality disorders among 230 adult relatives of their probands. The data on the first 34 families analyzed indicate that 32 percent of relatives from the stage I sample, 26 percent of relatives from the stage II sample, and 13 percent of relatives from the control sample suffered from DSM–III–R-defined personality disorders.

Such personality disorders are characterized by inflexible and maladaptive traits that cause the person significant impairment in social and/or occupational functioning. Conduct disorder in childhood and paranoid personality disorder in adulthood are examples of personality disorder.

Not surprisingly, personality disorders occurred more frequently in alcoholic relatives (40 percent) than in nonalcoholic relatives (28 percent). Alcoholic and nonalcoholic relatives also differed in the kind of disorders they had. For example, cluster B disorders as defined by DSM–III–R (borderline, histrionic, and narcissistic personalities)^2^ occurred in 19 percent of the alcoholic relatives but only in 6 percent of the nonalcoholic relatives. Researchers currently are investigating whether these numbers represent true differences between alcoholic and nonalcoholic subjects.

### Comparison of DSM–III–R, DSM–IV, and ICD–10 Diagnoses

SSAGA’s structure also allows comparison of DSM–III–R-, DSM–IV-, and ICD–10-based diagnoses of abuse (called harmful use in ICD–10) and dependence within the same person. COGA investigators have analyzed the levels of agreement between these diagnostic systems for several drug-use disorders (e.g., abuse of alcohol, cannabinols, and opiates) in 1,922 subjects. The proportion of subjects diagnosed with either abuse or dependence was similar for the three systems, and the subjects generally received the same labels in the three diagnostic systems. The level of agreement was high, especially for dependence diagnoses, whereas diagnoses of abuse or harmful use were inconsistent, as has been reported previously (Schuckit et al. 1994).

Scientists now are examining subgroups within the COGA sample (e.g., comparing men and women, or people who have received inpatient treatment and those who never have received such treatment) to identify factors that may contribute to the discordance among diagnostic schemes. This process hopefully will improve the genetic analyses by identifying the most robust diagnostic criteria for alcohol dependence in probands and their family members.

### Male-Female Differences in Alcohol-Related Symptomatology

COGA researchers have begun to examine gender differences in alcohol-related symptoms, including their prevalence and severity. Some statistically significant gender differences have been found for the symptoms that make up the DSM–III–R diagnostic criteria for alcohol dependence (Bierut et al. 1995). For example, more female alcoholic probands reported alcohol-related gastrointestinal problems (e.g., stomach disease or vomiting blood due to drinking) than did male probands (9.2 percent versus 5.4 percent). Furthermore, female probands were more likely than male probands to report arguments while drinking (76 percent versus 71 percent) and concern about being an excessive drinker (71 percent versus 66 percent). Whereas these first analyses have focused only on alcohol-related symptoms, researchers now also are exploring possible gender differences (e.g., gender-specific phenotypes) using other domains (e.g., blood chemistry, electrophysiology, and cognitive functioning) of the COGA database.

### Evaluation of Alcoholism Subtypes

Several researchers previously have attempted to define subtypes of alcoholics that have common characteristics with respect to drinking patterns and drinking consequences (e.g., Jellinek 1960; Cloninger et al. 1981; Babor et al. 1992). Investigators have begun to assess the validity of these subtypes using data from COGA. These studies focus on two of the subtypes posited by Jellinek (1960), the gamma and delta subtypes, as well as on the multivariate A and B subtypes proposed by Babor and colleagues (1992).

COGA investigators have begun to categorize subjects who meet DSM–III–R criteria for alcohol dependence according to Jellinek’s typology. Preliminary findings indicate that out of 1,045 subjects analyzed to date, 341 (33 percent) met the criteria for gamma alcoholism, 204 (20 percent) did so for delta alcoholism, and 94 (9 percent) fell into both categories (see Cadoret et al. 1994). However, 406 subjects (39 percent) did not meet the criteria for either subtype.

COGA researchers also have analyzed their proband sample with respect to the characteristics underlying the typology of Babor and colleagues. This study identified two proband groups that parallel the A and B subtypes (Schuckit et al. 1995*c*). Once COGA’s initial sample collection is complete, additional studies will compare the relative usefulness of these and other typologies for predicting the etiology and transmissibility of alcoholism and investigate additional biochemical and electrophysiological characteristics of various subtypes.

### Latent-Class Analysis of Alcohol-Use Symptomatology

COGA researchers not only have assessed the validity of existing typologies but also have attempted to identify new alcoholism subtypes based on the alcohol-related symptoms described in existing diagnostic systems (Bucholz et al. 1995*b*). Using a statistical technique that seeks to identify unobserved, or latent, classes or groups among the variables evaluated, the researchers identified four groups of drinkers from a sample of 2,551 adult biological relatives of alcoholic patients. The four classes included:

- Nonproblem drinkers (37.8 percent of all male subjects analyzed and 50 percent of all female subjects analyzed)
- Heavy drinkers with a persistent desire to stop and who experience tolerance and blackouts (31.1 percent of males and 28.8 percent of females)
- Heavy drinkers with social problems and some health and emotional problems (19.9 percent of males and 14.6 percent of females)
- Severely affected alcoholics with physiological dependence, an inability to abstain, and significant health and emotional problems (11.2 percent of males and 6.7 percent of females).

The study found little evidence for a separate alcohol abuse category as currently defined by DSM–III–R. Hazardous use (i.e., alcohol consumption patterns that may result in psychological or physical/medical harm to the user), in contrast, was ubiquitous, even among the nonproblem drinkers. Current findings based on the COGA sample of patients’ biological relatives suggest a bimodal distribution of drinkers. One part of the distribution describes symptomatic but non-dependent drinkers, whereas the other part of the distribution is characterized by the same, but more severe, symptoms. Thus, when alcoholism symptoms alone are used to construct a phenotype, the clinical state does not appear to be differentiated by distinctive symptom profiles (e.g., social consequences versus biological consequences) but rather falls along a continuum of severity.

The different analyses of the COGA data set presented above clearly point to the multifaceted nature of the clinical condition called “alcoholism.” Therefore, as a complex behavior, alcoholism may be the end product of several different genes located on different chromosomes rather than a single gene disorder such as the medical conditions of Huntington’s disease or Duchenne’s dystrophy. Thus, the successful hunt for the multiple genetic bases of alcoholism may require that several phenotypes representing different aspects of the syndrome be considered. For example, the latent-class phenotypes may be useful for examining the genetic aspects of the severity of alcoholism, whereas phenotypes based on the medical consequences of alcoholism may provide clues for understanding male/female variations in vulnerability to the biological consequences of chronic alcohol use. As a result, the genetic puzzle of alcohol dependence will be solved, but probably only one small piece at a time.

## Footnotes

2 A person with borderline disorder exhibits pervasive instability of mood and interpersonal relationships, those with histrionic disorders show excessive emotionality and attention-seeking behavior, and those with narcissistic disorder have a grandiose sense of self-importance and a constant need for attention.

## References

[b7-arhw-19-3-228] Babor TF, Hoffman M, DelBoca FK, Hesselbrock V, Meyer RE, Dolinsky ZS, Rounsaville B (1992). Types of Alcoholics, I: Evidence for an empirically derived typology based on indicators of vulnerability and severity. Archives of General Psychiatry.

[b8-arhw-19-3-228] Baron M, Endicott J, Ott J (1990). Genetic linkage in mental illness: Limitations and prospects. British Journal of Psychiatry.

[b9-arhw-19-3-228] Bierut LJ, Bucholz KK, Reich TR (1995). Alcohol dependence predicts medical problems equally in men and women. Alcoholism: Clinical and Experimental Research.

[b10-arhw-19-3-228] Bucholz K, Cadoret R, Cloninger CR, Dinwiddie S, Hesselbrock V, Nurnberger J, Reich T, Schmidt I, Schuckit M (1994). A new, semi-structured psychiatric interview for use in genetic linkage studies: A report of the reliability of the SSAGA. Journal of Studies on Alcohol.

[b11-arhw-19-3-228] Bucholz KK, Hesselbrock VM, Shayka JJ, Nurnberger JI, Schuckit MA, Reich TR (1995a). Reliability of individual diagnostic criterion items for psychoactive substance dependence and impact on diagnosis. Journal of Studies on Alcohol.

[b12-arhw-19-3-228] Bucholz KK, Heath AC, Reich T, Hesselbrock VM, Kramer J, Nurnberger JI (1995b). Subtyping alcoholism using latent class analysis: 1. Data from relatives in a multicenter family study of alcoholism. Alcoholism: Clinical and Experimental Research.

[b13-arhw-19-3-228] Cadoret R, Kramer J, Crowe R Personality disorders among the relatives of alcoholic probands.

[b14-arhw-19-3-228] Cloninger CR, Bohman M, Sigvardsson S (1981). Inheritance of alcohol abuse: Cross-fostering analyses of adopted men. Archives of General Psychiatry.

[b15-arhw-19-3-228] Hesselbrock V, Hesselbrock M, Cloninger CR, Begleiter H (1990). Social and behavioral factors which may affect the genetic expression of alcoholism. Genetics and Biology of Alcoholism.

[b16-arhw-19-3-228] Jellinek EM (1960). Alcoholism: A genus and some of its species. Canadian Medical Association Journal.

[b17-arhw-19-3-228] Maziade M, Roy MA, Fournier JP (1992). Reliability of best-estimate diagnosis in genetic linkage studies of major psychoses. American Journal of Psychiatry.

[b18-arhw-19-3-228] Rice JP, Reich TR, Bucholz KK, Fishman R, Rochberg N, Hesselbrock VM, Nurnberger JI, Schuckit MA, Begleiter H (1995). Comparison of direct interviews and family history diagnoses of alcohol dependence. Alcoholism: Clinical and Experimental Research.

[b19-arhw-19-3-228] Schuckit MA, Hesselbrock V, Tipp J, Anthenelli R, Bucholz K, Radziminski S (1994). A comparison of DSM–III–R, DSM–IV and ICD–10 substance use disorders diagnoses in 1922 men and women subjects in the COGA study. Addiction.

[b20-arhw-19-3-228] Schuckit MA, Anthenelli RM, Bucholz KK, Hesselbrock VM, Tipp J (1995a). The time course of development of alcohol-related problems in men and women. Journal of Studies on Alcohol.

[b21-arhw-19-3-228] Schuckit MA, Hesselbrock VM, Tipp J, Nurnberger JI, Anthenelli RM, Crowe RR (1995b). The prevalence of major anxiety disorders in relatives of alcohol dependent men and women. Journal of Studies on Alcohol.

[b22-arhw-19-3-228] Schuckit MA, Tipp J, Smith TL, Shapiro E, Hesselbrock V, Bucholz K, Reich T, Nurnberger JI (1995c). An evaluation of Type A and Type B alcoholics. Addiction.

[b23-arhw-19-3-228] Weissman MM, Merikangas KR, John K, Wickramaranate P, Prusoff BA, Kidd KK (1986). Family-genetic studies of psychiatric disorders. Archives of General Psychiatry.

# Research article: NEUROPHYSIOLOGICAL COMPONENT

**Published:** 1995

**Authors:** Bernice Porjesz, Henri Begleiter

**Affiliations:** Bernice Porjesz, Ph.D., is an assistant professor and Henri Begleiter, M.D., Ph.D., is a professor of psychiatry and neuroscience in the Department of Psychiatry, State University of New York Health Science Center, Brooklyn, New York

The neurophysiology component of COGA measures probands’ electrical brain activities with noninvasive scalp electrodes using two techniques: the EEG and event-related potentials (ERP’s). ERP’s are brain waves that are elicited in response to specific stimuli (e.g., a light or sound) and provide sensitive measures of cognitive brain activity. ERP’s consist of several waves, or components. The component most frequently studied in alcohol research is P300, a positive wave that occurs about 300 milliseconds after the stimulus. The height of the wave (i.e., the amplitude) is related to the significance of a stimulus.

Several studies have found that among other ERP aberrations, abstinent alcoholics exhibit a reduced P300 amplitude in response to a stimulus (for review, see Porjesz and Begleiter 1993). In addition, subjects at risk for alcoholism, particularly sons of alcoholic fathers, exhibit low P300 amplitudes even prior to alcohol exposure (Begleiter et al. 1984; for review, see Polich et al. 1994). And a recent meta-analysis of the entire literature on P300 in high-risk individuals concluded not only that “the P300 component is a useful investigative tool in this context but also that it may have predictive value as an index of vulnerability for alcoholism when well-designed paradigms are used to elicit ERPs” (Polich et al. 1994, p. 67).

The reduced P300 amplitude is a consistent finding that seems to characterize people at risk for alcoholism and which may serve as a phenotypic marker for alcoholism. Furthermore, twin studies have provided evidence that P300 characteristics are heritable (Polich and Burns 1987; O’Connor et al. 1994). COGA provides a unique opportunity to assess the value of P300 as a phenotypic marker for alcoholism.

## COGA’s Neurophysiology Component

Laboratories at all six COGA centers collect EEG and ERP information on members of the stage II and control families. The experiments also assess P300 characteristics. The control families are randomly selected and represent the population at each COGA site. Control subjects with alcoholism or other psychiatric illnesses are not excluded so that the prevalence of these disorders corresponds to that in the general population.

For each of the ERP-related experiments, researchers have examined the consistency of the findings across the six COGA sites. For example, in experiments measuring P300 in response to light stimuli, the wave forms and the P300 amplitudes did not differ significantly among sites (Cohen et al. 1994). Similar results were obtained for a number of other experiments (Alexander et al. 1994; Kuperman et al. 1995). Because of these similarities, data from all study sites can be analyzed together.

## Results From COGA’s Neurophysiological Studies

### P300 in Women and Men

Most investigations to date have demonstrated reduced P300 amplitudes only in male alcoholics but have not studied female alcoholics. Data from the COGA project, however, indicate that female alcoholics also manifest a diminution in P300 amplitude, although not to the same degree as male alcoholics (Porjesz et al. 1995). In addition, high-risk daughters as well as sons of male alcoholics exhibit low P300 amplitudes.

### P300 in Stage II Families

COGA researchers compared the distribution of P300 amplitudes in subjects over age 16 from control families with the P300 amplitudes of members of densely affected stage II families (i.e., based on direct interviews using SSAGA at least three first-degree relatives were alcoholic) (Porjesz et al. in press). The control sample included 687 people from 163 randomly selected families. The stage II sample was composed of 1,276 subjects from 219 families. After statistical analyses, only P300 amplitudes at least 2 standard deviations (SD’s) below the mean of each group were considered an abnormal trait. The P300 distributions of age- and sex-matched subjects were compared. All differences detected were statistically significant (*p* < 0.0001).

All stage II subjects combined had a significantly lower mean P300 amplitude than the control subjects, and more stage II subjects than control subjects had abnormally low P300 amplitudes (table 1). The investigators obtained similar results when they compared alcoholic stage II subjects with alcoholic controls. Even among nonalcoholic stage II subjects, the mean P300 amplitude was significantly lower than among nonalcoholic controls, indicating that the family density for alcoholism may be more important in determining a person’s P300 amplitude than a diagnosis of alcoholism in that person. The data also indicated that among the stage II sample, alcoholic subjects had significantly lower P300 amplitudes than nonalcoholic subjects, whereas no significant differences existed between alcoholic and nonalcoholic control subjects. In addition, male alcoholics in the stage II sample had significantly lower P300 amplitudes than female alcoholics.

Compared with offspring of male nonalcoholic control subjects, offspring of male alcoholics from the stage II sample had significantly lower P300 amplitudes, which in turn fell more frequently at least 2 SD’s below the mean (17.5 percent compared with 2.5 percent in the controls). These offspring will be followed in longitudinal studies through adolescence and early adulthood, because recent findings (Berman et al. 1993; Hill et al. 1995) indicate that low P300 amplitudes in young boys predict future substance abuse as adolescents.

## Conclusions

The data from the COGA project confirm that low P300 amplitudes may be a useful phenotypic marker for the risk of alcoholism. Recent studies have found that electrophysiological aberrations in alcoholics and people at risk also exist in ERP measures other than P300 (Porjesz and Begleiter 1994). The combination of several electrophysiological measures may represent a more specific phenotypic marker for a predisposition to alcoholism than reduced P300 alone.

## References

[b24-arhw-19-3-228] Alexander JE, Polich J, Bloom FE, Bauer LO, Kuperman S, Rohrbaugh J, Morzorati S, O’Connor SJ, Porjesz B, Begleiter H (1994). P300 from an auditory oddball task: Inter-laboratory consistency. International Journal of Psychophysiology.

[b25-arhw-19-3-228] Begleiter H, Porjesz B, Bihari B, Kissin B (1984). Event-related potentials in boys at risk for alcoholism. Science.

[b26-arhw-19-3-228] Berman MS, Whipple SC, Fitch RJ, Noble EP (1993). P3 in young boys as a predictor of adolescent substance abuse. Alcohol.

[b27-arhw-19-3-228] Cohen HL, Wang W, Porjesz B, Bauer BO, Kuperman S, O’Connor SJ, Rohrbaugh J, Begleiter H (1994). Visual P300: An interlaboratory consistency study. Alcohol.

[b28-arhw-19-3-228] Hill SY, Steinhauer S, Lowers L, Locke J (1995). Eight-year longitudinal follow-up of P300 and clinical outcome in children from high-risk for alcoholism families. Biological Psychiatry.

[b29-arhw-19-3-228] Kuperman S, Porjesz B, Arndt S, Bauer LO, Begleiter H, Cizadlo T, O’Connor SJ, Rohrbaugh J (1995). Multi-center N400 ERP consistency using a primed and unprimed word paradigm. Electroencephalography and Clinical Neurophysiology.

[b30-arhw-19-3-228] O’Connor SJ, Morzorati S, Christian JC, Li TK (1994). Heritable features of the auditory oddball event-related potential: Peaks, latencies, morphology and topography. Electroencephalography and Clinical Neurophysiology.

[b31-arhw-19-3-228] Polich J, Burns T (1987). P300 from identical twins. Neuropsychologia.

[b32-arhw-19-3-228] Polich J, Pollock VE, Bloom FE (1994). Meta-analysis of P300 amplitude from males at risk for alcoholism. Psychological Bulletin.

[b33-arhw-19-3-228] Porjesz B, Begleiter H, Nixon SJ, Hunt WA (1993). Neurophysiological factors associated with alcoholism. Alcohol-Induced Brain Damage.

[b34-arhw-19-3-228] Porjesz B, Begleiter H Neurophysiology in individuals at high risk for alcoholism.

[b35-arhw-19-3-228] Porjesz B, Begleiter H, Litke A, Bauer LO, Kuperman S, O’Connor SJ, Rohrbaugh J Neurophysiological phenotypes in alcoholism.

[b36-arhw-19-3-228] Porjesz B, Begleiter H, Litke A, Bauer LO, Kuperman S, O’Connor SJ, Rohrbaugh J Visual P3 as a potential phenotypic marker for alcoholism: Evidence from the COGA national project.

# Research article: BIOCHEMICAL MARKERS

**Published:** 1995

**Authors:** Ting-Kai Li

**Affiliations:** Ting-Kai Li, M.D., is a professor of medicine and biochemistry at the School of Medicine, Indiana University, Indianapolis, Indiana

Over the years, several biochemical and molecular measures have been reported to be associated with alcoholism and therefore may serve as potential trait markers of alcoholism. These markers include certain proteins, called MNS’s, found on red blood cells; the red blood cell esterase D enzyme, which exists in several molecular forms with slightly different characteristics (i.e., is polymorphic); and the activities of the enzymes platelet monoamine oxidase (MAO) and platelet adenylate cyclase. Researchers are studying these and other potential markers in the COGA families and the control families. The large size and familial distribution of the COGA sample offer researchers an unprecedented opportunity to test whether these characteristics in fact are trait markers of alcoholism.

Analysis of data collected to date has yielded the following conclusions:

- Esterase D polymorphism and MNS’s genotypes could not distinguish between alcoholic and nonalcoholic people in more than 2,000 subjects from both COGA and control families.
- Linkage analyses for more than 570 sibling pairs in the COGA families revealed no evidence that alcohol dependence was linked to regions on chromosome 13 containing the esterase D gene and the region on chromosome 4 containing the MNS’s genes. Similarly, no evidence existed for linkage of several genetic markers located near these genes to alcohol dependence.
- Analyses of MAO activity levels in more than 2,000 subjects found that women as a group had higher MAO levels than men. Furthermore, alcoholic males and females had lower MAO activity than nonalcoholic males and females. Finally, the sons and daughters of alcoholics exhibited lower MAO activity than the sons and daughters of nonalcoholics.
- In contrast to previous studies, MAO activity levels did not distinguish between subtypes of alcoholism (e.g., primary alcoholism versus secondary alcoholism or type A versus type B alcoholism). Lower MAO activity generally was associated, however, with earlier onset and more severe forms of alcoholism.
- Current smoking significantly affected MAO activity; after controlling for smoking, the correlation between an alcoholism diagnosis and MAO activity was no longer significant. MAO activity is, however, still subject to major genetic influences and thus may continue to be useful as a genetic marker of psychopathology underlying alcoholism.

In summary, the biochemical marker studies to date have provided convincing evidence that esterase D and MNS’s are not useful trait markers of alcoholism. In addition, the studies have found that platelet MAO activity is heavily influenced by both gender and smoking. Previous study results that had not corrected for these effects therefore should be viewed with caution. Researchers currently are investigating the utility of MAO as a marker of personality and temperament characteristics that might increase risk for alcoholism. They also are examining the relationship of platelet adenylate cyclase activity to alcoholism.

# Research article: ALCOHOL CHALLENGE COMPONENT

**Published:** 1995

**Authors:** Marc A. Schuckit

**Affiliations:** Marc A. Schuckit, M.D., is a professor of psychiatry at the University of California, San Diego, School of Medicine, and director of the Alcohol Research Center, San Diego Veterans Affairs Medical Center, San Diego, California

The search for genes that contribute to the risk for a disorder as complex and heterogeneous as alcohol dependence would benefit from the identification of more homogeneous subgroups of alcoholic families. One marker that might characterize such a population could be a less intense psychological and physiological response to alcohol (e.g., lower subjective feelings of intoxication and hormone levels). Such a low-level response (LR) is four times more common in sons of alcoholics than in control subjects and thus may help to predict future alcoholism in young men (Schuckit and Smith in press *a*).

To investigate this hypothesis, scientists are studying 18- to 30-year-old sons and daughters of alcoholics from the potentially most informative families in an alcohol-challenge experiment at the COGA center in San Diego, California. Although these subjects drink alcohol, they are not alcohol dependent. The experiment consists of three parts: an acclimation session, an alcohol-challenge session during which the participants receive 0.9 milliliter of alcohol per kilogram of body weight, and a placebo session. Directly after consuming the beverage and then every 15 to 30 minutes for the next 3 hours, the subjects are evaluated for changes in their subjective feelings of intoxication, levels of body sway,^3^ and levels of certain hormones.

During the past 4 years, the researchers have tested more than 100 COGA subjects, recently publishing the data obtained from the first 20 pairs of Caucasian sons of alcoholic fathers (Schuckit et al. in press *b*). These analyses showed that, compared with the control group, the subjects with a family history of alcoholism had significantly reduced responses to alcohol consumption, as measured by alcohol-induced changes in the level of the hormone cortisol and by subjective feelings of intoxication, with trends in the same direction for all other measures.

The alcohol-challenge protocol recently has been expanded to include alcohol-related changes in ERP’s and to test appropriate women in COGA families. The goal is to identify families in which one or more subjects exhibit LR. Researchers then could focus on these families during linkage analyses as a possible subgroup of the COGA population with a relatively unique phenotype. This approach might help to study more homogeneous samples in the linkage analyses.

## References

[b37-arhw-19-3-228] Schuckit MA, Smith TL An 8-year follow-up of 450 sons of alcoholics and controls. Archives of General Psychiatry.

[b38-arhw-19-3-228] Schuckit MA, Tsuang JW, Anthenelli RM, Tiggs JE, Nurnberger JI Alcohol challenges in young men from alcoholic pedigrees and control families. Journal of Studies on Alcohol.

# Research article: MOLECULAR GENETICS

**Published:** 1995

**Authors:** Howard J. Edenberg

**Affiliations:** Howard J. Edenberg, Ph.D., is a professor in the departments of biochemistry and molecular biology and medical and molecular genetics, Indiana University School of Medicine, Indianapolis, Indiana

Many genes, in addition to environmental factors, contribute to a person’s risk for alcoholism, and the contribution of any one gene to this risk seems limited. Consequently, a study of the genetics of alcoholism should be comprehensive and not focus on any one potential candidate gene.

From its inception, the COGA project has envisioned searching the entire genome for genes affecting the risk of alcoholism. This approach offers the highest probability of finding the relevant genes. This systematic screening process also includes proposed candidate genes, using highly polymorphic markers (see below) either within or adjacent to the genes. Once researchers locate a region that appears to contribute to the risk for alcoholism, they will examine additional markers and potential candidate genes within that region to confirm the initial finding. This appears to be the most powerful approach to identifying the genetic basis of a complex disease such as alcoholism.

## Polymorphic Markers

During the past few years, and largely as a result of the Human Genome Project, researchers have identified thousands of markers called microsatellites and mapped their locations on the human genome. These markers are sites on the DNA at which short stretches of two, three, or four nucleotides (i.e., building blocks of DNA) are repeated several times. The repeats are highly polymorphic—that is, each person has a characteristic number of repeats for each marker site that can be easily identified and which is inherited from both parents. Polymorphism allows researchers to track the transmission of the microsatellites across successive generations of a family. The microsatellites serve as markers for the chromosomal regions in which they reside. To find chromosomal regions and genes influencing alcoholism, investigators look for certain microsatellites that are co-inherited with the disease across multiple generations.

A biochemical technique called polymerase chain reaction (PCR) can help identify a person’s alleles for each marker site. Using small amounts of the person’s DNA, scientists can establish the length of each marker allele, which depends on the number of repeats present at that site. By comparing this genetic information with phenotypic information (e.g., whether a person is alcoholic), researchers can determine whether any region of the genome (i.e., any marker) is inherited in a manner consistent with its containing a gene affecting the risk for alcoholism.

## COGA’s Genotyping Project

The first phase of the COGA project involves determining the genotypes of 994 subjects from 106 families, using a set of markers that are spaced, on average, about 20 cM apart, with more than 200 markers in all. To ensure the accuracy of such a large-scale genotyping effort, the markers chosen are highly polymorphic (i.e., are most likely to differ among people), and their length can be easily and reliably determined. Many markers that would be useful in a small project present too many difficulties when scaled up to determine nearly 1,000 genotypes.

To improve the accuracy with which allele sizes are determined, two researchers independently examine each allele and enter their data into a computer. After comparing the data, any discrepancies are marked and reevaluated. As before, the two “readers” determine the allele size independently. These new data again are compared and only those alleles for which both readers agree on a size are entered into the database for further analysis.

Initially, each subject’s genotype is determined without reference to family relationships and without knowing the subject’s phenotype. A second round of analysis then serves to identify allele patterns that do not conform with the basic laws of inheritance (i.e., a person has an allele that apparently has not been inherited from either the mother or father). These so-called non-Mendelian inheritances can arise from spontaneous mutations in the repeated sequences, laboratory mistakes, or errors in the stated family relationships. Data on apparent non-Mendelian inheritances can be entered into blank pedigrees for further examination and corrections when necessary.

## Outlook

The COGA investigators have solved many of the technical problems of analyzing micro-satellites in large numbers of DNA samples. To increase the speed, efficiency, and accuracy of this formidable genotyping project, they have automated many steps, for example, by using DNA sequencing machines to determine allele sizes. Other measures have included the design of data systems that allow for error checking at multiple stages. With these measures in place, data generation on the COGA sample now is under way.

# Research article: DATA ANALYSIS

**Published:** 1995

**Authors:** John P. Rice

**Affiliations:** John P. Rice, Ph.D., is a professor of mathematics in psychiatry in the Department of Psychiatry, Washington University School of Medicine, St. Louis, Missouri

Advances in establishing a linkage map of the human genome have enabled researchers to rapidly map the genes underlying diseases that are caused by a single gene and which are familially transmitted according to the basic Mendelian laws of inheritance (e.g., cystic fibrosis or Huntington’s disease). Scientists now are focusing on identifying genes that contribute to common, complex diseases, such as alcoholism. A person’s susceptibility to these disorders depends on the interactions of multiple genetic and environmental factors. Twin, adoption, and family studies support the hypothesis of a strong genetic component for alcoholism. The next step is to identify specific disease-susceptibility genes.

The linkage of a disease phenotype with genetic markers can be used to determine the location of the disease-susceptibility genes. This process, however, can be difficult and time consuming. For example, with Huntington’s disease, a genetically simple disease, linkage with a genetic marker first was reported in 1983, yet it took researchers 10 years to identify the gene involved. For traits or diseases that are controlled by several genes, it may be much more difficult to identify the susceptibility genes.

An important component to detecting susceptibility genes is adequate analysis of the data provided by genome screens and phenotypic analyses. COGA researchers have studied several approaches to analyzing the genetic data.

One approach to identifying disease-susceptibility genes is to study the association of the disease phenotype with candidate genes. A critical consideration in this approach is to select appropriate controls. Analyses of a diverse (i.e., heterogeneous) population can lead to spurious associations. For example, if a subgroup of a population has blond hair and blue eyes, the association of these traits might be interpreted as evidence that the genes for these traits are linked. To avoid such problems, COGA researchers have analyzed data using the so-called haplotype relative risk method, which compares alcoholic subjects and their parents and uses parental alleles that have not been transmitted to the offspring as the control sample. This method also can be used for genes with more than two alleles (Rice et al. 1995).

Valid data analysis methods also are critical for evaluating data from genomic linkage analyses using simple sequence repeat polymorphisms. One approach to studying traits with a complex genetic basis, such as alcoholism, is to compare marker alleles in affected sibling pairs (or more generally, affected relative pairs) to identify shared markers. Other statistical methods can be used in multigenerational families to produce positive results even if the analysis is based on a simplified genetic model. Researchers are using simulation experiments to identify statistical approaches suitable for linkage analyses of traits that are determined by the additive effect of several susceptibility genes combined with environmental factors. The results of these simulations will guide the specific analytic approaches to evaluating data from the COGA genomic screening project.

## References

[b39-arhw-19-3-228] Rice JP, Neuman RJ, Hoshaw SL, Daw EW, Gu C (1995). TDT with covariates and genomic screens with Mod Scores: Their behavior on simulated data. Genetic Epidemiology.
